# Sexual attraction with pollination during feeding behaviour: implications for transitions between specialized strategies

**DOI:** 10.1093/aob/mcad178

**Published:** 2023-11-14

**Authors:** Ryan D Phillips, Björn Bohman, Rod Peakall, Noushka Reiter

**Affiliations:** Department of Environment and Genetics and the Research Centre for Future Landscapes, La Trobe University, Melbourne, Victoria 3086, Australia; Ecology and Evolution, Research School of Biology, The Australian National University, Canberra, ACT 2600, Australia; Royal Botanic Gardens Victoria, Science Division, Corner of Ballarto Road and Botanic Drive, Cranbourne, VIC 3977, Australia; Kings Park and Botanic Garden, The Botanic Garden and Parks Authority, West Perth, WA 6005, Australia; Ecology and Evolution, Research School of Biology, The Australian National University, Canberra, ACT 2600, Australia; Department of Plant Protection Biology, the Swedish University of Agricultural Sciences, Lomma 23422, Sweden; School of Molecular Sciences, The University of Western Australia Crawley, WA 6009Australia; Ecology and Evolution, Research School of Biology, The Australian National University, Canberra, ACT 2600, Australia; Ecology and Evolution, Research School of Biology, The Australian National University, Canberra, ACT 2600, Australia; Royal Botanic Gardens Victoria, Science Division, Corner of Ballarto Road and Botanic Drive, Cranbourne, VIC 3977, Australia

**Keywords:** Sexual deception, nectar, pollination, evolution, Orchidaceae

## Abstract

**Background and Aims:**

Understanding the origin of pollination by sexual deception has proven challenging, as sexually deceptive flowers are often highly modified, making it hard to resolve how any intermediate forms between sexual deception and an ancestral strategy might have functioned. Here, we report the discovery in *Caladenia* (Orchidaceae) of sexual attraction with pollination during feeding behaviour, which may offer important clues for understanding shifts in pollination strategy.

**Methods:**

For *Caladenia robinsonii*, we observed the behaviour of its male wasp pollinator, *Phymatothynnus* aff. *nitidus* (Thynnidae), determined the site of release of the sexual attractant, and experimentally evaluated if the position of the attractant influences rates of attempted copulation and feeding behaviour. We applied GC-MS to test for surface sugar on the labellum. To establish if this pollination strategy is widespread in *Caladenia*, we conducted similar observations and experiments for four other *Caladenia* species.

**Key Results:**

In *C. robinsonii*, long-range sexual attraction of the pollinator is via semiochemicals emitted from the glandular sepal tips. Of the wasps landing on the flower, 57 % attempted copulation with the sepal tips, while 27 % attempted to feed from the base of the labellum, the behaviour associated with pollen transfer. A similar proportion of wasps exhibited feeding behaviour when the site of odour release was manipulated. A comparable pollination strategy occurs in another phylogenetically distinct clade of *Caladenia*.

**Conclusions:**

We document a previously overlooked type of sexual deception for orchids involving long-distance sexual attraction, but with pollination occurring during feeding behaviour at the labellum. We show this type of sexual deception operates in other *Caladenia* species and predict that it is widespread across the genus. Our findings may offer clues about how an intermediate transitional strategy from a food-rewarding or food-deceptive ancestor operated during the evolution of sexual deception.

## INTRODUCTION

Macroevolutionary studies of floral evolution have provided compelling evidence that many plant lineages have repeatedly undergone shifts in pollination strategy, often between different groups of animal pollen vectors (Van der Niet and Johnson, 2012). The evolution of novel functional traits for the attraction of pollinators and their correct positioning for pollen transfer is often required during these shifts (Phillips *et al.*, 2020*b*). These functional trait changes may involve floral colour, shape, odour, and availability or type of food reward (e.g. Muchhala, 2007; Shuttleworth and Johnson, 2009; Peakall *et al.*, 2010; Jersáková *et al.*, 2012). In most cases it seems unlikely that a concurrent set of beneficial mutations will arise that can underpin all of the necessary changes in floral traits to instantaneously exploit a new pollinator group. A more plausible scenario is that a lineage passes through an intermediate stage of double function (Stebbins, 1970), where a plant attracts both the original and the new pollinator group, with pollinator-mediated selection driving further refinements (Van der Niet *et al*., 2014). However, due to trade-offs in the floral traits required to attract different pollinators, traits at this intermediate stage may be suboptimal for both strategies and reduce the likelihood of pollinator switching (Muchhala, 2007; Phillips *et al.*, 2020*b*). This raises the questions, what form do these intermediate stages take and how do they function?

Pollination by sexual deception is a geographically widespread phenomenon that has evolved multiple times across the Orchidaceae, spanning at least four continents (Kullenberg 1961; Stoutamire, 1974; Singer, 2002; Vereecken *et al.*, 2012; Cohen *et al.*, 2021; Ackerman *et al.*, 2023), and is documented from two known cases outside the orchids (Johnson and Midgley, 1997; Ellis and Johnson, 2010; Vereecken *et al*., 2012). In this highly specialized pollination strategy, plants typically attract the males of a single species of pollinator to their flower through the mimicry of specific insect sex pheromones [Schiestl *et al.*, 1999, 2003; Bohman *et al.*, 2014, 2016, 2017; Xu *et al.*, 2017; Peakall *et al.*, 2020; but see Phillips *et al*. (2017) and Schatz *et al.* (2021) for examples with multiple pollinator species]. In orchids, the flowers often possess insect-like structures that may act as visual and tactile mimicry of the females, as well as floral structures that aid the precise positioning of the pollinator (Peakall, 1990; Blanco and Barboza, 2005; Phillips *et al.*, 2014*b*; De Jager and Peakall, 2016). Phylogenetic evidence suggests that in some cases sexual deception has evolved from lineages pollinated via food deception (Inda *et al.*, 2012; Weston *et al.*, 2014; Cohen *et al*., 2021) or by insects using flowers as shelter sites (Vereecken *et al.*, 2012). Given that the floral traits of sexually deceptive flowers are often very different to those of related species that use other pollination strategies, how transitions to or from sexual deception arise is an intriguing question.

The diverse Australian orchid genus *Caladenia* (>350 species) is a promising system to investigate the evolution of pollination by sexual deception. Thus far, *Caladenia* appears to be unique worldwide in that while pollination by sexual deception is a taxonomically widespread pollination strategy (>150 species), the pollination strategies of food deception, and more rarely of nectar reward, also operate (Stoutamire, 1983; Faast *et al.*, 2009; Phillips *et al.*, 2009, 2017, 2020*a*, 2021; Swarts *et al.*, 2014; Reiter *et al.*, 2018, 2019*a*, *b*). *Caladenia* also shows remarkable variation in floral traits, ranging from large, brightly coloured species, which include Australia’s largest orchid flowers, through to diminutive species with an insectiform labellum (Backhouse, 2018). Based on current knowledge, all sexually deceptive *Caladenia* exploit male thynnine wasps for pollination (e.g. Stoutamire, 1983; Swarts *et al.*, 2014; Phillips *et al.*, 2009, 2017). On the other hand, relatively little is known about the types of pollinators used by the non-sexually deceptive *Caladenia* species. For three species, solitary bees are now known to be the primary pollinators (Faast *et al*., 2009; Reiter *et al.*, 2019*a*; Phillips and Batley, 2020), while *Caladenia drummondii*, a phylogenetically distinct species, is pollinated by nectar-feeding pompilid wasps (Phillips *et al.*, 2021). However, the recent discovery of pollination by male thynnine wasps that show food-seeking behaviour (Reiter *et al.*, 2018, 2019*b*; Phillips *et al*., 2020*a*), rather than being sexually attracted to the flower, raises the possibility that transitions in pollination strategies could occur without a shift in pollinator family (Reiter *et al*., 2018). Indeed, while some *Caladenia* pollinated by nectar-seeking thynnine wasps have just one primary pollinator species, the pollinator genera are sometimes shared with sexually deceptive *Caladenia* (see Swarts *et al.*, 2014; Reiter *et al.*, 2018, 2023).

**Fig. 1.**
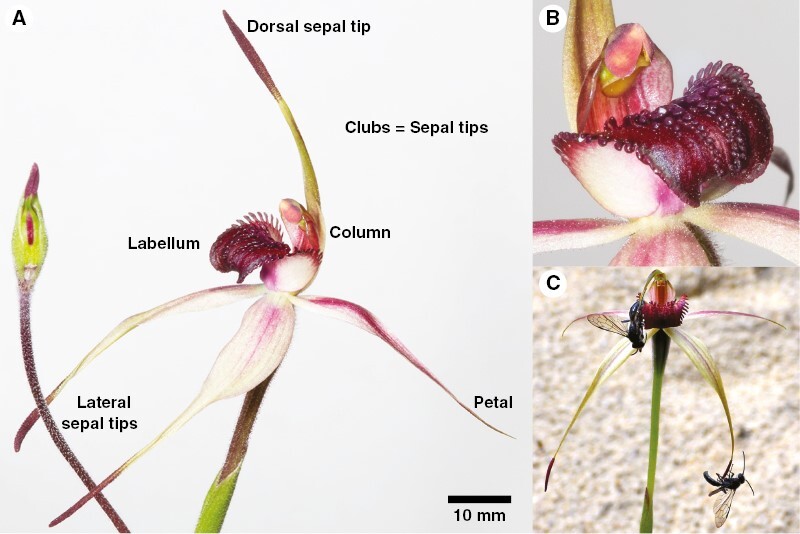
*Caladenia robinsonii* showing the flower, nectar and attempted copulation by *Phymatothynnus* aff. *nitidus*. (A) A flower with floral parts labelled, (B) a close-up of the labellum, showing liquid that has been exuded onto the upper surface, and (C) male *P*. aff. *nitidus* attempting copulation with the clubs of a *C. robinsonii* flower. These clubs are the source of sexual attractant. Images (A) and (B) by Rod Peakall, and image (C) by Noushka Reiter.

While undertaking pollinator surveys for a conservation programme for the endangered orchid *Caladenia robinsonii*, two preliminary and unexpected observations prompted this detailed investigation of the pollination of *C. robinsonii*: (1) while males of a single species of thynnine wasp, *Phymatothynnus* aff. *nitidus*, exhibited sexual behaviour with the flower, some individuals appeared to exhibit feeding behaviour at the flower; and (2) visible nectar was evident on the labella of some flowers in cultivation and in the wild. *Caladenia robinsonii* belongs to the *C. reticulata* complex, a sexually deceptive group of orchids that typically exploit closely related, often taxonomically cryptic species of male *Phymatothynnus* thynnine wasps (Swarts *et al.*, 2014; Reiter *et al.*, 2023). Like most other members of the *C. reticulata* complex, *C. robinsonii* has dull-coloured red and cream flowers with a dark maroon labellum tip, and glandular, swollen sepal tips (Fig. 1; Backhouse, 2018) – all features that in *Caladenia* are typically associated with pollination by sexual deception (Stoutamire, 1983; Phillips *et al*., 2017).

Our first aim was to test whether pollen removal and deposition is associated with sexual or feeding behaviour in *C. robinsonii*. Our second aim was to better understand the process of pollinator attraction. We addressed this aim via a series of experiments that answered the following questions: (1) Is long-distance attraction of pollinators mediated by sexual cues? (2) Which part of the flower produces the sexual attractant? (3) Is surface sugar present on the upper surface of the labellum, even when no nectar is visible? Following confirmation that the glandular sepal tips (hereafter referred to as ‘clubs’) are the sole source of sexual attractant, we experimentally manipulated the position of the clubs to test if odour emission from clubs rather than the labellum facilitates more frequent feeding behaviour, which in *C. robinsonii* is typically associated with pollen removal and deposition. Having demonstrated that *C. robinsonii* combines sexual attraction with pollination occurring during feeding behaviour, our third aim was to test if this strategy is more taxonomically widespread by applying similar observations and experiments across a representative set of other *Caladenia* species.

## METHODS

### Study species

*Caladenia robinsonii* is listed as Endangered under the *Environment Protection and Biodiversity Conservation Act* 1999, and is now only known from one natural site, which is a small remnant (6 ha) within a suburban area on the east side of Port Phillip Bay, Victoria, Australia (Backhouse *et al*., 1999). This site has been heavily supplemented with propagated plants of *C. robinsonii*. Propagated plants have also been introduced at two other sites (see Supplementary Data S1). Historically, *C. robinsonii* is believed to have been endemic to the Mornington Peninsula on the eastern edge of the greater metropolitan area of Melbourne, in southeastern Australia (Backhouse, 2018), most of which has now been cleared for housing and farming. The vegetation communities that *C. robinsonii* favoured are believed to be the coastal heathlands that were bordered by woodland (Threatened Species Scientific Committee, 2016).

In *C. robinsonii*, flowering occurs during late September and early October, with typically one flower (occasionally two) produced per scape (Backhouse, 2018). Most individuals have a dark red labellum, with red and cream-streaked sepal and petals (Backhouse, 2018; Fig. 1). Other than the labellum, the tepals are linear-lanceolate, with the sepals narrowing to swollen terminal glands (clubs) held distal from the centre of the flower. The labellum is stiffly articulate and is capable of tipping towards the column if a small amount of force is applied. Examination of flowers during warm conditions revealed glistening on the upper surface of the labellum, and sometimes visible liquid that appeared to be exuding from the top of the calli (Fig. 1). Based on our observation that none of ~200 plants in a pollinator-free glasshouse set any seed (N. Reiter, pers. obs.), we conclude that *C. robinsonii* requires a pollen vector to achieve pollination.

While there is little information available on the life history of *P*. aff. *nitidus*, some generalities can be drawn from the biology of thynnine wasps (Thynnoidea: Thynnidae). Females burrow underground where they lay eggs on scarab beetle larvae, with the wasp larvae consuming the beetle larva prior to pupation (Ridsdill-Smith, 1970). Upon emerging from the ground, the flightless female crawls to a prominent position and releases a sex pheromone to attract the volitant males (Peakall, 1990; Schiestl *et al*., 2003; Bohman *et al*., 2014, 2017), which then compete to mate with the female (Alcock, 1981). The successful male then carries the female *in copula* to a food source, which is often nectar but also includes exudates from psyllids and scale insects (Burrell, 1935; Phillips *et al*., 2009; Brown and Phillips, 2014). Recently, male thynnine wasps have been reported to exhibit feeding behaviour on *Caladenia* flowers that have no visible nectar droplets, though the presence of surface sugar was confirmed with GC-MS (e.g. Reiter *et al.*, 2018, 2019*b*). In a previous study of possible food sources of male *P.* aff. *nitidus*, swabs revealed pollen grains of *Leptospermum myrsinoides* and an unidentified *Acacia* on the bodies of all 19 individuals tested (Reiter, 2019). While *P*. aff. *nitidus* has been observed feeding on nectar from the flowers of *L. myrsinoides*, no direct observations of feeding have been made on *Acacia*. In the case of the *Acacia*, the wasps may be feeding from extrafloral nectaries, as seen with some other species of thynnine wasp (Bernhardt, 1987).

### *General methods for studying pollinators in* C. robinsonii

Pollinator observations were undertaken with the baiting method used for sexually deceptive orchids (Stoutamire, 1974; Peakall, 1990). In this method, picked orchids are moved to a new position in the landscape, leading to a rapid response from sexually deceived male pollinators (Peakall, 1990). Due to the extreme rarity of *C. robinsonii*, the flowering plants used for pollinator observations and experiments were sourced from propagated material (see Table 1). Thus, whole flowering plants in pots, rather than picked specimens, were used. These plants were symbiotically propagated following the methods of Reiter *et al.* (2016) using the fungus *Serendipita australiana*, the mycorrhizal associate sourced from the remaining wild population of *C. robinsonii* (Reiter *et al*., 2020). All baiting experiments were conducted at reserves where the pollinator was known to be common, but the orchid is not naturally found: Langwarrin Reserve and the Royal Botanic Gardens Victoria (RBGV) at Cranbourne (Supplementary Data S1). It was confirmed using DNA barcoding of the mitochondrial DNA (mtDNA) *COI* region that all populations belonged to the same wasp species (Data S1).

**Table 1.** The presence of surface sugar on the labellum and the source of sexual attractant in *Caladenia robinsonii* and other species of *Caladenia*. NA, not applicable.

Species	Amount of surface sugar (µg, mean ± s.e.)	Feeding behaviour documented	Length of feeding time (s)	Source of sexual attractant	Copulation	Reference
*Caladenia abbreviata*	Not measured	Yes, rarely	No data	Clubs	Clubs	Phillips and Peakall (2018*a*)
*Caladenia attingens*	0	No	NA	Labellum, clubs	Labellum, clubs	Present study
*Caladenia crebra*	0	No	NA	Primarily clubs	Labellum	Present study
*Caladenia infundibularis*	0	Yes, rarely	1.8 ± 0.8	Clubs	Clubs, labellum	Present study
*Caladenia procera*	8.0 ± 4.5	Yes, rarely	1.7 ± 0.7	Clubs	Clubs	Present study
*Caladenia robinsonii*	219.4 ± 38.7	Yes, occasionally	3.6 ± 0.7	Clubs	Clubs	Present study
*Caladenia tentaculata*	0	No	NA	Labellum, clubs	Labellum, clubs	Peakall and Beattie (1996); Reiter *et al.* (2018)

### *Pollinator behaviour in* C. robinsonii

**Fig. 2.**
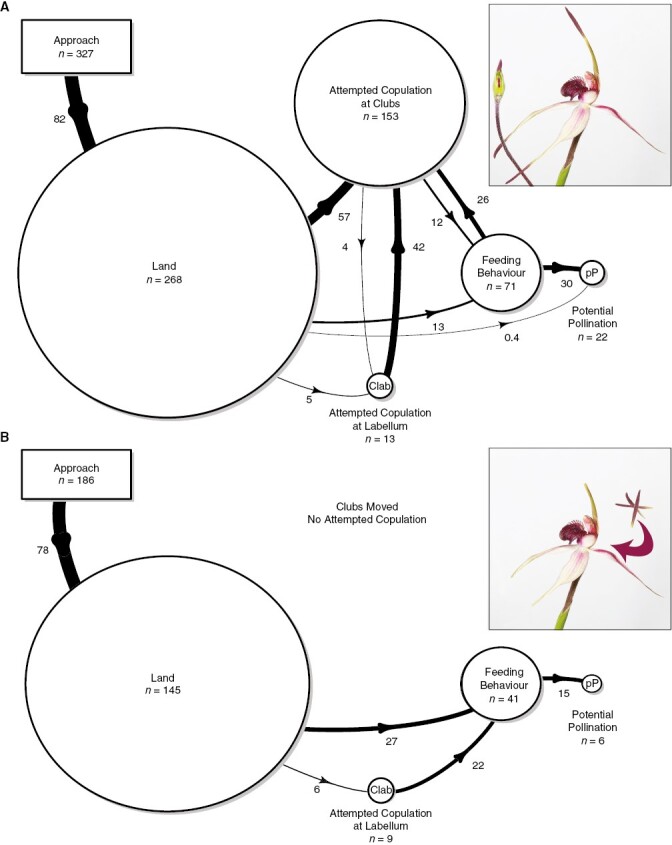
A summary of pollination behaviour on *Caladenia robinsonii* illustrated using flow diagrams. Numbers represent the proportion of individuals transitioning from one behaviour to the next. Size of the circles represents the total number of individuals exhibiting that behaviour. (A) A summary across the control flowers for all experiments (the natural state), and (B) the experimental treatment when the source of the sexual attractant is moved under the labellum. A = approach only, L = landed, Cclubs = copulated with clubs, Clab = copulated with labellum, F = attempted to feed, pP = potential pollination (contacted the column). In the flow diagrams, data on the order of feeding and copulatory behaviour were extrapolated from the subset of observations where these details were recorded.

Natural pollinator behaviour was documented by combining data across two 5-min baiting trials with five different flowers, and across the control treatments of the experiments described below. Across all experiments, we recorded the following behaviour for each individual wasp: (1) if they landed on the flower; (2) if they attempted copulation with the flower and with which floral part (attempted copulation defined as either probing with the tip of the abdomen, sustained downward curling of the abdomen or genitalia exposed); (3) if there was any feeding behaviour as evident by the head positioned close to the flower, and extension of the labium and maxillae (rather than the mandibles) onto the surface of the labellum; and (4) if they contacted the column and whether pollen was removed or deposited. We also recorded if pollen removal/column contact was associated with sexual behaviour, feeding behaviour or neither. For a subset of observations, one of us recorded the directionality of any switches from feeding to sexual behaviour (or vice versa) and, with the aid of a stopwatch, the length of time spent attempting to feed from the flower. For each of the 17 flowers observed, we calculated the percentage of responding wasps exhibiting attempted copulation, feeding behaviour and contact with the column, and calculated the overall mean, median and interquartile range across flowers. We prepared flow diagrams to visualize the overall patterns of pollinator behaviour observed at *C. robinsonii* (for intact flowers and the treatment in the clubs under the labellum experiment – see below). In these diagrams (see Fig. 2), we partitioned pollinator behaviours into six categories: approach (A), land (L), attempted copulation at the clubs (Cclubs), attempted copulation at the labellum (Clab), feeding behaviour (F) and potential pollination (pP) when column contact was observed.

### *Long-distance pollinator attraction in* C. robinsonii

Due to odour-based attraction in other sexually deceptive orchids, we predicted that the wasp pollinators would be readily able to locate hidden flowers. To test if pollinators could be attracted to the flower by chemical cues alone, a single bait flower was first presented either inside or outside of an opaque box (~10 cm taller than the plants, with an open top to allow odour dispersal) for a duration of 6 min. This was followed by presentation for a further 6 min in the alternative position at a different baiting site. We repeated this experiment for four flowers, alternating the start order of inside/outside for the four flowers.

### *The source of sexual attractant in* C. robinsonii

Flowers were dissected into column, labellum, clubs and the remaining floral display. Each tissue was then pinned to its own bamboo skewer using a dressmaker’s pin with a black head, and held in position at the top of the skewer using a small piece of plasticine. Preliminary choice tests with all four tissue types indicated that the clubs were the primary source of sexual attractant. Thereafter, we used sequential choice tests (following Phillips *et al.*, 2013) where the column, labellum and floral display were first presented 50 cm apart in a line perpendicular to any wind direction for 2 min before introducing the clubs for an additional 2 min. For each dissected flower, five trials were conducted ~100 m apart, with the position of the floral parts in the line randomized in each trial. Six replicate experiments were conducted, each using different flowers. Due to the drastic difference in attractiveness of the various floral parts, no statistical test was needed.

### Site of sexual attractant and pollinator behaviour

In most sexually deceptive orchids, copulation occurs with the labellum, which is the primary source of the sexual attractant. However, given that in *C. robinsonii* the distally held sepal clubs are the source of attractant, and that we observed pollinators exhibiting feeding behaviour, we designed an experiment to test if emission of the sexual attractant in closer proximity to the labellum leads to reduced feeding and therefore less frequent contact with the reproductive structures. In this experiment, the three sepal clubs were excised and pinned under the labellum of the treatment flowers. The intact control flowers were not manipulated, except for a 2-mm incision that was made on the base of the dorsal sepal (which is obscured from pollinator view; as per de Jager and Peakall, 2016), to control for the incisions made to remove the clubs of the treatment flowers. Two observers worked simultaneously, recording pollinator behaviour over two 5 min trials (~50 m apart) for either the treatment or the control flowers. Six replicate experiments were conducted, each using a different pair of control and treatment flowers, with the observers switching between recording data for the control or treatment.

We tested for statistical differences in the proportion of responding wasps that contacted the column, and the proportion of responding wasps that either fed or copulated with the flower by using generalized linear mixed models (GLMMs). These analyses were run in *lme4* (Bates *et al*., 2015) using the function glmer with a binomial distribution. We treated experimental replicate (1–6) as a random factor in recognition that a different pair of flowers was used for each experimental replicate and the different environmental conditions that wasps were exposed to across experimental replicates. The statistical significance of the variable ‘site of odour release’ was tested by comparing the model with and without this variable using likelihood-ratio tests (using the ‘anova’ function). Marginal means for the fixed effect were calculated and plotted using the *ggeffects* package in R (Lüdecke, 2018).

### *Visitation of other insect species to* C. robinsonii

Following our observation that some flowers of *C. robinsonii* produce visible nectar, we designed an experiment to test if other potential insect visitors show feeding behaviour and could potentially act as pollinators. In an attempt to avoid sexually attracting the usual male thynnine wasp pollinator, we simultaneously presented multiple pots of cultivated plants (with one or two flowers per scape), containing a total of 30 flowers, whose clubbed sepal tips were removed (de-clubbed) just prior to use. We baited with 30 flowers, since similar large numbers have proven effective at attracting food-seeking pollinators of other *Caladenia* species (Reiter *et al*., 2018, 2019*a*, *b*; Phillips *et al*., 2020*a*; Phillips and Batley, 2020). For each trial, we first baited for 6 min with the group of 30 de-clubbed flowers, before introducing a single intact flower of *C. robinsonii* for another 6 min to confirm that pollinators were active. Six replicate experiments were conducted, each consisting of three trials, and using a different control flower per experiment.

### Surface sugar sampling, derivatization and GC-MS analysis

We sampled ten flowers of *C. robinsonii* for surface sugar, each from a different plant, based on the methods of Reiter *et al*. (2018), which uses a derivatization method adapted from Lisec *et al*. (2006). Sampling was conducted at 20 °C. For each flower, three 5-µL droplets of an aqueous solution of ribitol (internal standard, 0.20 mg mL^–1^) were added using a glass syringe on to the upper surface of the labellum. This quantity of liquid only covered a portion of the labellum, so the drop was positioned centrally where the feeding behaviour of pollinators was focused. The aqueous extract was subsequently collected with microcapillary tubes (5 µL) and immediately transferred to an insert (150 µL) inside a 2-mL GC vial, with the three extracts for each flower combined in the same vial. Extracts were stored in a −20 °C freezer until analysis. Quantification of glucose, fructose and sucrose was achieved by comparison of peak areas of total ion chromatograms (TICs) of samples with the known amount of the internal standard ribitol. The response factors for the respective monosaccharides (glucose and fructose) and disaccharide (sucrose) sampled and the internal standard were measured and included in the calculation of the amounts of analytes.

### *Is the pollination strategy of* C. robinsonii *found in other* Caladenia?

To determine if the pollination strategy of *C. robinsonii* is found more widely across the genus, we extended our pollinator observations, floral dissection choice experiments and surface sugar analysis to a strategic selection of *Caladenia*. We targeted four *Caladenia* species representing different species complexes that were morphologically well defined (e.g. Hopper and Brown, 2001) and phylogenetically distinct (Peakall *et al.*, 2021). These four species have previously been confirmed to be pollinated by sexually attracted male thynnine wasps: (1) *C. attingens* subsp. *attingens* is primarily pollinated by *Thynnoides* sp. (Phillips *et al*., 2017); (2) *C. crebra* is pollinated by *Campylothynnus flavopictus* (Bohman *et al.*, 2017); (3) *C. infundibularis* is pollinated by *Thynnoides* sp. (Phillips *et al*., 2017); and (4) *C. procera* is pollinated by *Zaspilothynnus nigripes* (Phillips *et al.*, 2009).

For each species, we quantified pollinator behaviour (as above) at solitary bait flowers across multiple trials of 3-min duration. Across the observations, we used five to seven different bait flowers per species, with a maximum of 15 trials per flower. Due to the fleeting nature of some nectar-feeding behaviour in these species, recordings by a human observer were supplemented with video using a Panasonic HC-V750M camcorder.

**Fig. 3.**
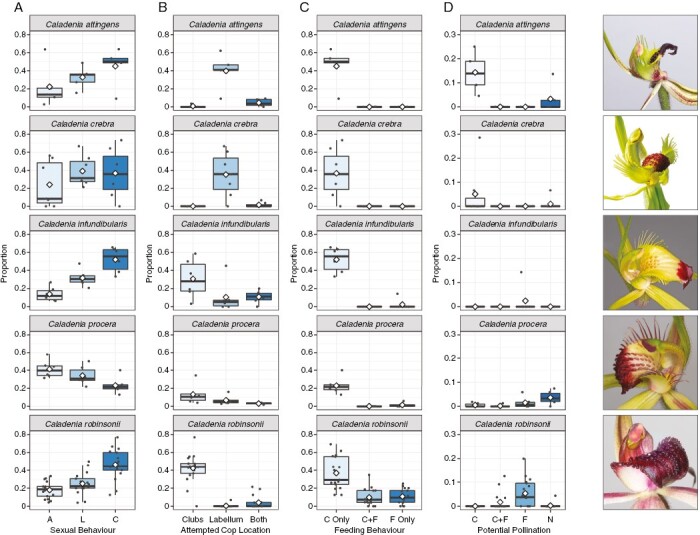
Summary of pollinator behaviour for each of the five study species. Column 1 – the proportion of individuals exhibiting copulatory behaviours per flower, with behaviours expressed in mutually exclusive categories: A = approached without landing; L = landed without exhibiting further behaviour; C = landed then copulated. Column 2 – the proportion of pollinators copulating with different floral parts. Column 3 – the proportion of pollinators engaging in copulatory and/or feeding behaviour: C only = copulation; C + F = copulation and feeding; F only = feeding. Column 4 – the proportion of individuals that potentially pollinated the flower (contact with the column) for different behaviours: C = copulation; C + F = copulation and feeding; F = feeding; N = no visible copulatory or feeding behaviour. Note, due to the low proportion of individuals potentially pollinating the flower (graphs in column 4), this axis is on a different scale to the graphs in columns 1–3. White diamonds represent the mean. Images by Rod Peakall.

To facilitate a qualitative graphical comparison across the other study species (*C. attingens*, *C. crebra*, *C. infundibularis* and *C. procera*) with *C. robinsonii*, we classified our pollinator observations with a focus on four different aspects. (1) *Sexual attraction*: here we used three mutually exclusive categories, approach only (A), land only (L) and attempted copulation (C). (2) *Attempted copulation location*: classified as either at the clubs (Cclubs), labellum (Clab), or copulating with both the clubs and labellum (Both). (3) *Feeding behaviour*: here subdivided into three mutually exclusive categories of copulation only without any prior or subsequent feeding behaviour (C), copulation and feeding behaviour irrespective of the order of these two behaviours (CF) and feeding behaviour without attempted copulation (F). (4) *Potential pollination* (pP): here, we subdivided the behaviours associated with potential pollination (as indicated by observed column contact) into the mutually exclusive categories of attempted copulation without feeding behaviour (C), attempted copulation and feeding behaviour (CF), feeding behaviour only (F), and no visible copulatory or feeding behaviour (N). See Fig. 3 and legend for further details.

Following the same methodology as used for *C. robinsonii*, for *C. attingens*, *C. crebra*, *C. infundibularis* and *C. procera* we undertook floral dissections to determine the source of the attractant. The only minor change was for *C. attingens*, where initial trials revealed that odour was produced from the labellum as well as the clubs. In this case, we baited with the column and floral remains for the first 3 min, before adding both the clubs and the labellum for an additional 3 min. Location details and the source of bait flowers are given in Supplementary Data S2. While none of these additional study species had been observed by us to produce nectar, or reported to do so in the literature, given our findings in *C. robinsonii*, we sampled six individual flowers of each species for surface sugar as outlined above.

## RESULTS

### *Pollinator behaviour in* C. robinsonii

At intact control bait flowers, responding male wasps (*P.* aff. *nitidus*) showed the prolonged stereotyped zig-zagging approach flight that is typical of sexually deceived thynnine wasps tracking an odour plume (e.g. Stoutamire, 1983; Peakall, 1990). As seen in other orchids pollinated by sexual deception of a thynnine wasp that is locally common, wasps responded to the flowers in large numbers. A total of 327 responses were observed to the 17 intact control flowers (from observations of *C. robinsonii*, and the intact control flowers from experiments). A total of 155 wasps attempted copulation with the flower (mean, median and IQR of 46, 44 and 20 % of responding wasps, respectively; Fig. 2), of which 153 individuals attempted copulation with the clubs and 13 attempted copulation with the labellum.

A total of 71 wasps showed feeding behaviour on the flower (20, 20 and 11 % of responding wasps; Fig. 2), including from flowers where there were no visible nectar droplets. Typically, feeding behaviour began near the central calli, with the insect subsequently moving forward towards to the base of the column (Supplementary Data S3). For those individuals that were timed, the mean (±s.e.) period of feeding behaviour was 3.6 ± 0.7 s (*n* = 8). The order of feeding and copulatory behaviour was recorded for 40 wasps. In 17 of these (42.5 %) they only showed feeding behaviour, while in 12 (30 %) they attempted copulation before feeding, and in 11 (26.5 %) they fed before attempting copulation. Contact with the column was observed on 22 occasions (7, 7 and 6 % of responding wasps), with 21 of these associated with feeding behaviour, and none associated with attempted copulation.

Given that all baiting was conducted in nature reserves lacking *C. robinsonii*, we did not expect to observe wasps carrying pollen, unless they had previously visited our bait flowers. A total of 14 wasps were observed removing pollen. In each case, pollen was deposited on the dorsal surface of the wasp’s thorax. Three cases of pollination (pollen deposition and/or transfer) were observed when wasps of suitable size had moved down the labellum and close to the base of the column, at which point the weight shifts the hinged labellum, temporarily trapping them between the labellum and stigma on the column. At this point any pollinia on the wasp thorax was held in direct contact with the stigma. Subsequently, pollen removal occurred after the wasp thorax was first smeared with sticky stigmatic secretions and then was brought into contact with the edge of one or both pairs of pollinia lobes within the anther, as the wasp appeared to struggle to extricate itself from the flower. Any feeding behaviour appeared to be interrupted by the wasp’s temporary entrapment. Across all experiments, 16 males were seen carrying pollen of *C. robinsonii*.

### *Long-distance pollinator attraction in* C. robinsonii

A total of 44 *P. nitidus* male wasps responded to and contacted the flowers positioned within an open-ended box (*n* = 4 flowers, mean wasps = 11 ± 2.1 s.e.), compared to a total of 81 responding to and contacting the visible flowers presented outside of the box (*n* = 4 flowers, mean wasps = 20.2 ± 3.9). Thus, as predicted, the specific male wasp pollinator of *C. robinsonii* can readily locate hidden flowers via chemical cues. Interestingly, two cases of pollen deposition and three cases of pollen removal were observed during these experiments.

### *The source of sexual attractant in* C. robinsonii

The first phase of the sequential choice tests, which consisted of exposing the column, labellum and floral display from a single dissected flower, failed to attract any visits by *P*. aff. *nitidus*. By contrast, during the second phase, where the clubs were introduced, a total of 241 wasps were attracted. During this phase three individuals landed on the floral display and nine on the labellum but none landed on the column. The clubs were visited by 229 wasps (*n* = 6 flowers; mean = 38.2 ± 4.5 wasps per flower), of which 212 landed (mean = 35.5 ± 3.5 wasps per flower) and 14 attempted to copulate with the clubs (mean = 2.3 ± 1.0 wasps per flower).

### Site of sexual attractant and pollinator behaviour

Artificially moving the source of sexual attractant by pinning the clubs under the labellum neither altered the proportion of wasps that attempted to feed from the flower (median: under labellum = 22 %; control = 27 %; GLMM results: *P* = 0.137; Fig. 2; see Supplementary Data S4 for marginal means), nor the proportion of wasps that contacted the column (median: under labellum = 0 %; control = 7 %; *P* = 0.276). However, with the odour-producing clubs effectively concealed out of reach from the pollinator, a significant reduction in the number of wasps attempting to copulate with the flower was observed (median: under labellum = 0 %; control = 44 %; *P* < 0.001), with only six attempting copulation with the tip of the labellum. By contrast, at the control flower, of the 60 wasps that attempted copulation, all did so with the clubs, while one also attempted copulation with the labellum. Overall, contrary to our prediction, no reduction in feeding behaviour was observed, while the overall attempted copulation rates plummeted, and virtually no attempted copulation was observed at the labellum itself.

### *Visitation of other insect species to* C. robinsonii

There was only one case of a species other than *P.* aff *nitidus* responding to *C. robinsonii*. In this instance, an individual of the introduced species *Apis mellifera* (Apidae) visited four de-clubbed flowers, feeding on the surface of the labellum of each flower for a prolonged period. While feeding, the tip of the labellum was tilted downward, preventing the bee from making contact with the flower’s reproductive structures. Interestingly, some *P.* aff. *nitidus* did respond to the de-clubbed flowers of *C. robinsonii*. However, in the second part of the experiment, when a single intact flower was presented simultaneously with the 30 de-clubbed plants, far more wasps responded to the single intact flower (*n* = 6 flowers; mean ± s.e for intact flower = 13 ± 1.9, 78 responses in total; mean for de-clubbed plants = 1.2 ± 0.7, seven responses in total). A total of 20 wasps exhibited feeding from the de-clubbed flowers (20, 20 and 14 % of responding wasps), while 14 wasps exhibited feeding from the natural control flower (19, 19 and 8 % of responding wasps). While no attempted copulation was observed with these de-clubbed flowers, wasps still showed odour-tracking behaviour.

### *Surface sugar measurements for* C. robinsonii

On average, 219.35 ± 38.67 µg (s.e.) (*n* = 10) of sucrose per sample was detected on the labellum lamina of *C. robinsonii*. Lesser amounts of fructose (1.16 ± 0.58 µg) and glucose (2.59 ± 1.58 µg) were also recorded.

### *Is the pollination strategy of* C. robinsonii *found in other* Caladenia?

As was the case in *C. robinsonii*, for all four additional study species, pollination (or potential pollination) only occurred when the male wasps faced the column with their head positioned near the base of the labellum and column. In this position, the weight shift tips the wasp forward and into contact with the stigma, momentarily entrapping the insect. Pollen deposition/pollen removal occurred as the wasp struggled to free itself from the flower. For the two species where attempts to feed were observed (*C. infundibularis* and *C. procera*), feeding was usually brief (<1.5 s) and commenced towards the base of the upper surface of the labellum. Feeding appeared to be interrupted when the male wasp became temporarily trapped (Supplementary Data S5 and S6).

#### Caladenia attingens

A total of 156 wasps responded, with 82 wasps attempting copulation (45, 50 and 5 % of responding wasps; *n* = 5 flowers; Fig. 3A). These consisted of 81 individuals attempting copulation with the labellum, and eight with the clubs. Twenty-four wasps contacted the column during attempted copulation with the labellum (18, 18 and 8 % of responding wasps, Fig. 3D). No wasps attempted to feed from the flower and no sugar was detected on the labellum (*n* = 6).

Floral dissections revealed that sexual attraction is only with the clubs and the labellum. When the clubs and labellum were presented to wasps simultaneously, 82 wasps responded to the clubs with 68 of them landing (11.3, 7 and 12.25 wasps landing per flower; *n* = 6 flowers), while 21 wasps responded to the labellum with 15 landing (2.5, 2.5 and 2.5 wasps landing per flower).

#### Caladenia crebra

A total of 113 wasps responded, with 46 wasps attempting copulation (37, 36 and 37 % of responding wasps; *n* = 7 flowers; Fig. 3A). All wasps that attempted copulation did so with the labellum, though two also attempted copulation with the clubs. Ten wasps made contact with the column (6, 0 and 7 % of responding wasps, Fig. 3D), and in nine instances this was associated with clearly recognizable copulatory behaviour (Fig. 3). No attempts to feed from the flower were observed and no sugar was detected on the labellum.

Floral dissections revealed that the clubs were the primary source of attractant. When other parts of the flower were presented in the absence of the clubs, five wasps responded to the labellum (three landing), and none to the column or floral display. When the clubs were introduced, 125 wasps responded to the clubs with 114 landing (19.0, 14 and 13.25 wasps landing per flower; *n* = 7 flowers), while only two wasps landed on the labellum, and there were no responses to the other floral parts.

#### Caladenia infundibularis

A total of 172 wasps responded, with 88 wasps attempting copulation (52, 56 and 21 % of responding wasps; *n* = 6 flowers; Fig. 3A). Of these, 68 wasps attempted copulation with the clubs and 40 with the labellum. Two wasps attempted to feed from near the base of the upper surface of the labellum (see Supplementary Data S4; feeding duration 1 and 2.5 s), which led to the only two cases of column contact (2, 0 and 0 % of responding wasps, Fig. 3D). No sugar was detected on the surface of the labellum of any of the six flowers tested.

Floral dissections revealed that the clubs were the only source of attractant. When other parts of the flower were presented in the absence of the clubs, no wasps responded. When the clubs were introduced, 122 wasps responded to the clubs with 93 landing (18.6, 17 and 4 wasps landing per flower; *n* = 6 flowers).

#### Caladenia procera

A total of 324 wasps responded, with 71 wasps attempting copulation (23, 21 and 4 % of responding wasps; *n* = 6 flowers; Fig. 3A), and six engaging in feeding behaviour (2, 1 and 2 % of responding wasps, Fig. 3C). A total of 45 wasps attempted copulation with the clubs, with 28 attempting to copulate with the labellum, and three attempting to copulate with the ovary or stem immediately below the flower. For the 20 wasps that contacted the column (6, 5 and 8 % of responding wasps, Fig. 3D), feeding behaviour was visible on six occasions, while column contact was never associated with copulatory behaviour (Fig. 3). Wasps attempted to feed from near the base of the upper surface of the labellum or the base of the column (see Supplementary Data S5; feeding duration 0.5, 1.5 and 3 s). On average, 8.0 ± 4.5 µg of sugar was detected on the surface of the labellum (*n* = 6), all of which was sucrose.

Floral dissections revealed that the clubs were the primary source of attractant. When other parts of the flower were presented in the absence of the clubs, two wasps responded to the labellum (none landing), and none to the column or floral display. When the clubs were introduced, 167 wasps responded to the clubs, with 87 of them landing (14.5, 11.5 and 10.25 wasps landing per flower; *n* = 6 flowers). During this phase of the experiment, two wasps approached the labellum without landing, and there were no responses to the other floral parts.

## DISCUSSION

Across the majority of known sexually deceptive orchids, an insectiform labellum is the focal point for male pollinators interacting with the flower. Pollen removal and deposition typically occurs during attempted copulation with the labellum (e.g. Coleman, 1929; Blanco and Barboza, 2005; Paulus, 2006; Phillips *et al.*, 2013; De Jager and Peakall, 2016; Cohen *et al.* 2021). However, the labellum is also centre stage in other types of sexually deceptive pollination. For example, in *Drakaea* the male pollinators attempt to fly-off with the labellum as if it was a female (Peakall, 1990). During this pre-copulatory behaviour, flexing of the hinge means that the wasp is tipped over and brought into contact with the reproductive structures. Although attempted copulation often occurs while wasps are temporarily trapped in this upside-down position, it is not strictly necessary for pollination (Peakall, 1990). In other cases of sexual deception, the pollinator is trapped in a galea following contact with the labellum and escapes via the stigma and anther (Phillips *et al.*, 2014b; Reiter *et al*., 2019*c*; Hayashi *et al.*, 2022). As such, while long-distance attraction by chemical cues is critical for pollination in sexually deceptive orchids, the final part of the pollination process can be achieved through copulatory behaviour, pre-mating behaviour or entrapment of the insect.

Here, we demonstrate for *C. robinsonii* an intriguing case of sexual deception that combines sexual attraction with pollination occurring during feeding behaviour. We show that this type of pollination extends to other *Caladenia* species representing different clades. In these cases of sexual deception, the labellum itself is neither insectiform nor the focus of attempted copulation. However, as is well documented in sexually deceptive orchids (Paulus and Gack, 1990, Singer, 2002; Peakall *et al*., 2010; Phillips *et al.*, 2014a, 2017; Cohen *et al*., 2021; Ackerman *et al.*, 2023), extreme pollinator specificity remains a characteristic of this form of sexual deception.

### *How does the pollination of* C. robinsonii *operate?*

Based on our observations and experiments we conclude that long-distance attraction in *C. robinsonii* is achieved via floral volatile sexual cues. Five lines of evidence from *C. robinsonii* support this conclusion: (1) despite more than 700 visits across our experiments and pollinator surveys at nearby reserves (Supplementary Data S1), only the males of a single species of thynnine wasp, *P.* aff. *nitidus*, were attracted to the flower; (2) male wasps were rapidly attracted to flowers concealed from view, often arriving within the first minute of experimental trials; (3) wasps approached both concealed and visible flowers via the typical zig-zag behaviour of insects tracking an odour plume originating from a point source; (4) rapid male wasp responses were a characteristic of all baiting experiments, attracting on average 21 wasp visitors per pair of 5-min trials; and (5) at control flowers, ~46 % of the visiting wasps (Fig. 2) attempted copulation with the flower (predominantly at the clubs). This behavioural evidence for long-distance sexual attraction of pollinators by floral volatiles in *C. robinsonii* matches observations in thynnine-pollinated sexually deceptive orchids, where the chemistry of pollinator attraction has been experimentally demonstrated in field bioassays with synthesized compounds (e.g. *Caladenia*, *Chiloglottis* and *Drakaea*; Schiestl *et al*., 2003; Peakall *et al*., 2010; Bohman *et al*., 2014, 2017, 2018, 2020; Xu *et al*., 2017).

After initial rapid arrival at the flower, the behaviour of the wasp pollinator at *C. robinsonii* differs fundamentally from well-known examples in the sexually deceptive genera *Caladenia*, *Chiloglottis*, *Disa*, *Drakaea* and *Ophrys*. In *C. robinsonii*, attempted copulation occurs almost exclusively with the clubs on the sepal tips, not the labellum. Further, contact with the reproductive structures only occurs during a switch to feeding behaviour on the labellum. While the feeding behaviour typically lasted only a few seconds, it serves to bring the wasp forward to where tilting of the labellum occurs, bringing the wasp into a position where pollination can occur. From our observations and experiments, at least three key features of *C. robinsonii* probably facilitate the switch to feeding behaviour. The labellum lacks an insectiform aggregation of calli, so there is no obvious visual or tactile stimulus to induce copulation, and the chemical cues for sexual attraction are exclusively emitted from the distal glandular clubs at the sepal tips. Further, the total surface sugar present on the labellum is at levels ~50 % higher than detected in any other *Caladenia* investigated so far (Phillips *et al*., 2021).

While many studies quantifying sugar use a refractometer to approximate the sugar concentration of nectar, out of necessity we have used surface washes, meaning that quantities are only broadly comparable with these other studies. Nonetheless, a mean of 223 µg of sugar per flower for *C. robinsonii* (equivalent of 22 % sugar for 1 µL of nectar) falls at the lower end of the reported range for sugar quantities in insect-pollinated orchids considered to provide a nectar reward (e.g. Galetto *et al*., 1997; Davies *et al*., 2005; Johnson, 2006; Albuquerque *et al*., 2021; Phillips *et al*., 2021; Jia and Huang, 2022). In multi-flowered food-deceptive orchids, the experimental addition of 400–500 µg per flower alters pollinator behaviour, with insects spending longer on flowers and visiting more flowers per inflorescence (e.g. Johnson *et al*., 2004; Jersáková and Johnson, 2006). For *C. robinsonii* it would be of interest to test if the presence of sugar encourages visitation to subsequent flowers, despite the use of sexual cues to attract the wasp and the struggle to extricate itself from the flower, which could act as a deterrent to future visits. While *C. robinsonii* is predominantly single-flowered, it may be possible to investigate this issue using experimental arrays.

### Prior evidence for a switch to feeding behaviour following sexual deception

The strategy of sexual deception with pollination during feeding behaviour appears not to have been previously documented in detail for any orchid. However, prior work in other orchid species supports its plausibility. In *Caladenia abbreviata*, another species that produces the sexual attractant from glands on the sepal tips, Phillips and Peakall (2018*a*) noted one instance of a wasp attempting to feed from the labellum. In *Ophrys*, Kullenberg (1961) showed that male bees attracted to flowers via sexual deception will switch to feeding if a sugar solution had been added to the flower. Interestingly, several orchid species that are pollinated by food-seeking pollinators exhibit a strong bias towards pollination by male rather than female insects (e.g. Bino *et al*., 1982; Sasaki *et al*., 1991; Johnson and Schiestl, 2016; Phillips *et al.*, 2021), suggesting that there could be other unproven cases where sexual cues contribute to odour-based long-distance attraction, although copulation with the flower does not occur.

A similar strategy to what we have documented in *C. robinsonii* is used in *Gorteria diffusa* (Asteraceae), a South African daisy pollinated by the bombyliid fly *Megapalpus nitidus* (Johnson and Midgley, 1997; Ellis and Johnson, 2010). In *G. diffusa*, pollination occurs in all populations by male and female flies feeding on nectar and pollen, but in some populations the presence of raised dark coloured spots on the ray florets leads to sexual behaviour from a proportion of the visiting male flies (Johnson and Midgley, 1997; Ellis and Johnson, 2010; De Jager and Ellis, 2012). Like *C. robinsonii*, in *G. diffusa* some male pollinators switch from mate-seeking to feeding behaviour (Johnson and Midgley, 1997), leading the authors to conclude that sexual deception and nectar reward can be combined as part of the same pollination strategy.

### *Is sexual deception with a switch to feeding behaviour widespread in* Caladenia?

All four additional *Caladenia* study species were strongly sexually attractive to their specific male thynnine wasp pollinators, with average rates of attempted copulation varying from 22 % in *C. procera* to over 40 % in the other species (Fig. 3). However, the location of attempted copulation varied, being predominantly on the labellum in *C. attingens* and *C. crebra*, compared with predominantly at the clubs, but sometimes at the labellum, or both, in *C. infundibularis* and *C. procera* (Fig. 3). No feeding behaviour was observed during pollinator visitation at *C. attingens* and *C. crebra*. Instead, contact with the column was associated with attempted copulation or grappling with the aggregated calli on the labellum (Fig. 3), which is the more typical pollinator behaviour in sexually deceptive orchids. By contrast, in *C. infundibularis* and *C. procera*, column contact and pollination occurred following a switch to feeding behaviour after initial sexual attraction. Thus, pollinator behaviour in these two species showed strong similarity to that observed in *C. robinsonii* (Fig. 3), despite belonging to different species complexes. As such, we predict this type of sexual deception will be taxonomically widespread in *Caladenia*.

No visible nectar was observed, nor has it been reported elsewhere for the four species. However, trace levels of sucrose were detected in *C. procera* but not the other three species. The finding of no detectable surface sugar on the labella of the six *C. infundibularis* flowers tested was unexpected, given that the two observed pollination events in this species were associated with a switch to feeding behaviour. However, it is important to note that we did not test the particular flower at which these observations were made. In the non-sexually deceptive *C. nobilis*, which appears to have a pollination strategy intermediate between food reward and food deception, pollination by its male thynnine wasp pollinator also occurs during feeding behaviour (Phillips *et al.*, 2020a). However, in this species, variability in the levels of detected surface sugar has been found, ranging from zero to minor levels of surface sugar (5.1 µg; Phillips *et al.*, 2020a). Thus, it is possible that male thynnine wasp feeding behaviour can be triggered by meagre amounts of surface sugar. Alternatively, other as yet unknown visual and/or olfactory cues, such as floral volatiles associated with food-rewarding flowers, might trigger the behavioural switch even in the absence of sugar.

Our work shows that sexually deceptive *Caladenia* exhibit a range of variation in the floral traits associated with their pollination. In the species we studied, measurements of surface sugar ranged from none, through to meagre amounts in *C. procera* (mean = 8 µg), to the much larger quantities in *C. robinsonii* (mean = 223 µg). Interestingly, our floral dissection experiments revealed two different patterns of odour release of the sexual attractant. In *C. attingens* and *C. crebra*, odour was emitted from the labellum and the clubs, as previously shown in *C. tentaculata* (Peakall and Beattie, 1996) and *C. williamsiae* (Phillips, 2017). Alternatively, in *C. robinsonii*, *C. infundibularis* and *C. procera*, the clubs were the sole source of sexual attractant, as previously reported for *C. abbreviata* (Phillips and Peakall, 2018*a*), *C. pectinata* (Phillips *et al*., 2013), *C. plicata* (Xu *et al*., 2017) and *C. xanthochila* (Reiter *et al*., 2023). While not experimentally studied here, *Caladenia* show pronounced interspecific variation in labellum morphology, which fall on a continuum from ‘insectiform’ – those with aggregated dark-coloured calli often prominently raised above the labellum lamina, such as in *C. crebra* (Fig. 3) – to ‘non-insectiform’ – those where the labellum lacks prominent aggregations of dark-coloured calli as in *C. procera*, *C. infundibularis* and *C. robinsonii* (Fig. 3). Based on present knowledge, pollination itself occurs either during attempted copulation with the labellum, or following a switch to feeding behaviour on the labellum. We predict that, rather than site of odour release, the combination of an insectiform labellum and an absence of surface sugar will characterise species where attempted copulation with the labellum is required for pollination.

### *Implications of dual behaviour for understanding pollination transitions in* Caladenia

The discovery that pollination is achieved during a switch to feeding behaviour from sexual deception in three different species complexes of *Caladenia* suggests that this could be an evolutionarily stable strategy, rather than a temporary transitional state between strategies using purely sexual deception or purely food-seeking behaviour. Nonetheless, such cases may offer vital new clues about how *Caladenia* has evolved such a wide variety of pollination strategies. Based on current evidence, pollination by food deception is almost certainly the ancestral state in *Caladenia* (Weston *et al.*, 2014), meaning that pollination during feeding behaviour is probably the ancestral condition to sexual deception, and that nectar production has evolved one or more times. Intriguingly, several recent studies have discovered that some non-sexually deceptive *Caladenia* species are primarily pollinated by one or two species of similar sized male thynnine wasps, with pollination occurring during feeding behaviour (Reiter *et al.*, 2018, 2019*b*; Phillips *et al.*, 2020*a*). While most of these orchid species show no visible nectar, trace sugar analysis has detected varying levels of surface sugar on the labellum ranging from 3 to 32 µg per flower (Reiter *et al*., 2018, 2019*b*; Phillips *et al*., 2020*a*). It is possible that the first step towards sexual deception occurred in such a species already exploiting male thynnine wasps as pollinators, with the production of even a partially effective sexual attractant potentially increasing the reliability of attracting the primary pollinator, while still relying on a switch to feeding behaviour for pollination itself. In such a case, the flower structure would already be adapted to that particular pollinator, with mutation(s) in just one or a few genes associated with semiochemical production holding the key to the transition toward sexual deception. The tissue specificity of semiochemical production and emission might also have been an important determinant in whether or not a full transition to sexual deception evolved.

Most sexually deceptive orchids (Schiestl *et al.*, 1999, 2003; Phillips *et al.*, 2013, 2014b), including some *Caladenia*, produce sexual attractants from an insectiform labellum (but see Singer, 2002), consistent with the hypothesis that this structure is key to both chemical and physical sexual mimicry. By contrast, in *C. robinsonii* and several other species [*C. procera* and *C. infundibularis* (this study); Phillips *et al.*, 2013; Xu *et al*., 2017; Phillips and Peakall, 2018*a*; Reiter *et al.*, 2023], the clubs on the sepal tips are the site of odour release. In *C. pectinata*, artificially moving the clubs under the labellum led to a significant increase in attempted copulation with the labellum, whereas attempted copulation is almost exclusively with the clubs in a natural flower (Phillips and Peakall, 2018*b*). In the present study of *C. robinsonii*, we also expected increased attempted copulation with the labellum, and decreased feeding behaviour – which would indicate a disadvantage to producing odour from the labellum. Interestingly, this was not the case. The only significant change in behaviour was an overall reduced copulation rate, as the wasps could no longer access the clubs. Thus, if variant *C. robinsonii* orchids produced odour from the labellum, pollination via a switch to feeding behaviour may neither be hindered nor enhanced with the current pollinator species. However, as evident in *C. pectinata* (Phillips and Peakall, 2018*a*), other pollinator species might behave differently, creating the potential for pollinator-mediated selection to drive the evolution of sexual mimicry involving copulation with the labellum.

### Do switches in behaviour operate in other forms of floral mimicry?

Our discovery that some sexually deceptive *Caladenia* species combine sexual attraction with pollination during a switch to feeding behaviour raises the question of whether a similar shift to feeding behaviour occurs in other cases of mimicry by flowers. Indeed, examination of the literature reveals this to be the case. For example, in the orchids *Epipactis helleborine* and *E. purpurata*, the flowers emit green-leaf volatiles that mimic damaged plant tissues to attract parasitic *Vespula* wasps searching for herbivorous caterpillar larvae to parasitize, with nectar instead offered as a reward for the deceived wasp (Brodmann *et al.*, 2008). In the Neotropical epiphyte orchid *Specklinia endotrachys*, *Drosophila* flies are lured to the flower by aggregation pheromones but are encouraged to linger on the flower through the provision of small quantities of nectar (Karremans *et al*., 2015). Among plants pollinated through brood site deception via false olfactory and visual cues, some species provide nectar to help position the insect appropriately for pollen removal and deposition (e.g. Meve and Liede, 1994; Bänziger, 2001). To test other plant species previously assumed to be pollinated via deception of pollinators it will be of interest to use our sensitive GC-MS-based method for the detection of trace levels of surface sugar (Reiter *et al*., 2018; this study). Given the evidence presented here, a rapid switch from other behaviours to feeding may be a widespread feature among insect pollinators.

## CONCLUSIONS

In our detailed study of thynnine wasp pollination of *C. robinsonii*, we confirm a new type of sexual deception for orchids. Unlike most sexually deceptive orchids, attempted copulation occurs with the odour-producing sepal tips, and pollination is not achieved during attempted copulation with an insectiform labellum. Instead, it requires a proportion of the responding male wasps to switch to feeding behaviour. This switching behaviour appears to be correlated with the presence of surface sugar on a non-insectiform labellum. Further, we show that sexual deception with pollination by feeding behaviour occurs in *C. infundibularis* and *C. procera*, representatives of other species complexes. In these two species, the labellum is also non-insectiform, and the clubs are the source of attractant. However, trace levels of sugar were only detected in *C. procera*, which may indicate that cues other than nectar or surface sugar trigger a shift to feeding behaviour. We predict that this new variation on the theme of sexual deception will be phylogenetically widespread across *Caladenia*.

Despite sexual deception being known as a widespread pollination strategy of the genus for ~50 years (Stoutamire, 1974), our discovery was unexpected. However, it is perhaps not surprising that it has been overlooked, particularly if cases such as *C. infundibularis*, where the switch to feeding behaviour is both fleeting and rare, are the norm. Expanded research into the pollination of other *Caladenia* species, in combination with a phylogenetic overlay of the traits we have identified in this study, promises to help understand the trajectory of floral evolution in *Caladenia*. As per the compelling arguments of Van der Niet (2020), our research highlights the importance of detailed studies of the natural history of pollination systems to help inform our understanding of evolutionary processes. Furthermore, our work on *C. robinsonii* is a poignant reminder of the surprising discoveries that can arise from programmes aimed at rescuing endangered species on the brink of extinction, and that with further losses of biodiversity we may irretrievably lose the potential for some ground-breaking evolutionary insights.

## SUPPLEMENTARY DATA

Supplementary data are available online at https://academic.oup.com/aob and consist of the following. S1: Surveying for *Phymatothynnus* aff. *nitidus*, the pollinator of *Caladenia*

*robinsonii.* S2: Location and voucher details for pollinator observations and floral dissections of species belonging to other species complexes of *Caladenia*. S3: Video of a thynnine wasp (*Phymatothynnus nitidus*) attempting to feed from the labellum of *Caladenia robinsonii*. S4: Effect plots illustrating marginal means for an experiment testing if the site of the sexual attractant affects wasp behaviour. S5: Video of a thynnine wasp (*Thynnoides* sp.) attempting to feed from the labellum of *Caladenia infundibularis*. S6: Video of a thynnine wasp (*Zaspilothynnus nigripes*) attempting to feed from the labellum of *Caladenia procera*.

mcad178_suppl_Supplementary_Data_S1

mcad178_suppl_Supplementary_Data_S2

mcad178_suppl_Supplementary_Data_S3

mcad178_suppl_Supplementary_Data_S4

mcad178_suppl_Supplementary_Data_S5

mcad178_suppl_Supplementary_Data_S6
