# Supplementary material for: Sexual attraction with pollination during feeding behaviour: implications for transitions between specialized strategies
Source: Ann Bot. 2023 Nov 14;133(2):273–86. doi: 10.1093/aob/mcad178 (PMC11005785; doi:10.1093/aob/mcad178)
Supplement: mcad178_suppl_Supplementary_Data_S1 [file mcad178_suppl_supplementary_data_s1.docx]

**SUPPLEMENTS**

**Supplement 1:** Surveying for *Phymatothynnus* aff. *nitidus*, the pollinator of *Caladenia robinsonii*.

*Methods*

Pollinator observations (see Table 1 for a list of sites) were undertaken using a modification of the baiting method used for sexually deceptive orchids (Stoutamire, 1974; Peakall, 1990). Here, we undertook baiting using pots of cultivated *C. robinsonii* that contained a total of six flowers. We conducted pollinator surveys between 2016 and 2018 at a total of 15 reserves on the Mornington Peninsula and the south-eastern suburbs of Melbourne. French Island was surveyed in 2016, the wild site (Site A) was surveyed in 2016 and 2017, and a further 11 reserves were surveyed in 2017 including RBGV Cranbourne (which was subsequently also surveyed in 2018). An additional two reserves were surveyed in 2018 (Table 1). Pollinator observations were conducted during October 2017 in sunny conditions exceeding 18**°** C, with light winds. At all sites baiting was conducted for 6-minute periods (103 baiting trials in total), moving to a new point in the landscape (approximately 100 metres apart) for each new baiting period. All floral visitors were recorded, along with whether they contacted the column, and removed or deposited pollinia. A total of 73 individuals of the pollinating species were captured for identification. Thynnine wasps were identified by Graham Brown at the Museum and Art Gallery of the Northern Territory using a series of unpublished keys to the Australian thynnine wasp fauna.

As there is evidence of cryptic species in some thynnine genera (Griffith et al. 2011), we undertook DNA barcoding to confirm the number of wasp species involved in pollination. This analysis was also used to confirm that the wasp species at the wild site was the same as those responding in behavioural studies undertaken in larger habitat remnants. Previous work in thynnine wasps has shown that the mtDNA *COI* sequence locus is highly effective at distinguishing between closely related species (Griffiths *et al*. 2011; Menz *et al*. 2015; Phillips *et al*. 2015). We sequenced 41 wasps, which were collected from ten of the sites between 2016 and 2017. This total includes wasps collected from the wild site during the 2016 and 2017 surveys (Supplement 1). DNA sequencing was undertaken following the methodology of Griffiths *et al*. (2011). A multiple sequence alignment was performed in Geneious Prime 2021.1.1 (https://www.geneious.com). Geneious was used to quantify the amount of genetic variation within the putative pollinator species based on percentage variation in the number of base pairs. In previous studies of thynnine wasps using the *COI* sequence locus, genetic variation across the geographic range of a species is almost always less than 5%, but typically less than 3% (Phillips *et al.* 2017).

*Results*

The only native floral visitors to *C. robinsonii* were males of the thynnine wasp (Tiphiidae) *Phymatothynnus* aff. *nitidus* (*N* = 435). DNA sequencing of the *COI* sequence locus (*N* = 41 individuals) revealed a maximum sequence divergence between individuals of only 1.022%, confirming that they all belonged to one species. In the surveys, *P.* aff. *nitidus* responded to flowers at 12 of the 15 survey sites, including at the only known wild site for *C. robinsonii*. In thynnine wasps only the males are volitant, with the winged male carrying the flightless female *in copula* and either feeding her directly or allowing her to forage on a food source (Alcock 1981). Of the male *P.* aff. *nitidus* visiting the flower, all were solitary rather than arriving *in copula* with a female. During the pollinator surveys, *P.* aff*. nitidus* was observed to remove (N = 15) pollen, and a further 18 individuals contacted the column.

*References*

Alcock J. 1981. Notes of the reproductive behaviour of some Australian thynnine wasps (Hymenoptera: Tiphiidae). *Journal of the Kansas Entomological Society* 54: 681–693.

Griffiths KE, Trueman JWH, Brown GR, Peakall R. 2011. Molecular genetic analysis and ecological evidence reveals multiple cryptic species among thynnine wasp pollinators of sexually deceptive orchids. *Molecular Phylogenetics and Evolution* 59: 195–205.

Menz MHM, Phillips RD, Anthony JM, Bohman B, Dixon KW, Peakall R. 2015. Ecological and genetic evidence for cryptic ecotypes in a rare sexually deceptive orchid, *Drakaea elastica*. *Botanical Journal of the Linnean Society* 177: 124–140.

Peakall R. 1990. Responses of male *Zaspilothynnus trilobatus* Turner wasps to females and the sexually deceptive orchid it pollinates. *Functional Ecology* 4: 159–167.

Phillips RD, Peakall R, Retter BA, Montgomery K, Menz MHM, Davis BJ, Hayes C, Brown GR, Swarts ND, Dixon KW (2015) Pollinator rarity as a threat to a plant with a specialized pollination system. *Botanical Journal of the Linnean Society* 179: 511-525.

Stoutamire W.P. (1974) Australian terrestrial orchids, thynnid wasps, and pseudocopulation. *American Orchid Society Bulletin* 43: 13–18.

**Table S1**: Details of survey sites for pollinators of *Caladenia robinsonii* used in the pollination study. For the wild site (Site A), precise location details have been withheld as a requirement of permits to work on threatened species. Translocations refer to plants that were symbiotically propagated at the Royal Botanic Gardens Victoria and then planted within similar suitable habitat of this species. *denotes DNA sequencing of the pollinator specimen was unsuccessful.

| **Site** | **Population status** | **Habitat** | **Latitude/longitude** | **Pollinator baiting** | **Voucher** |
| --- | --- | --- | --- | --- | --- |
| RBG Cranbourne | Translocation site | Heathy woodland | -38.1310°, 145.2736° | 2017-18 | W1556, W1557, W1533, W1534, W1535*, W1536 |
| Langwarrin | No plants present | Heathy woodland | -38.1745°, 145.1695° | 2017 | W1542, W1543* |
| Greens Bush | No plants present | Heathy woodland | -38.4115°, 144.9504° | 2017 | W1518, W1519, W1520, W1521, W1522, W1532, W1555 |
| Site A (details withheld) | Natural and Translocated site | Heathy woodland | -38.4°, 144.9° | 2016-2017 | W1514, W1515, W1516, W1517, W1434, W1435, W1436, W1438, W1439, W1440, |
| French Island | No plants present | Heathy woodland | -38.3660°, 145.3462° | 2016 | W1433, W1437 |
| Bald Hill | No plants present | Heathy woodland | -38.3499°, 145.0212° | 2017 | Pollinator not detected |
| Woods Bushland Reserve | No plants present | Heathy woodland | -38.2850°, 145.0793° | 2017 | W1523, W1524, W1525 |
| Private Property (details withheld) | No plants present | Heathy woodland | -38.4°, 144.9° | 2017 | W1539, W1540, W1541 |
| Site B (details withheld) | Translocation site (subsequently after baiting) | Heathy woodland | -38.4°, 144.9° | 2017 | W1537, W1538 |
| The Pines | No plants present | Heathy woodland | -38.1228°, 145.1775° | 2017 | W1529, W1530*, W1531 |
| Wooleys Beach | No plants present | Coastal Heathy Woodland | -38.3514°, 145.2163° | 2017 | Present, captured but not sequenced |
| Lornars Triangle | No plants present | Damp Woodland | -38.3514°, 145.1691° | 2017 | Pollinator not detected |
| Main Ridge Reserve | No plants present | Damp Woodland | -38.4137°, 144.9938° | 2017 | Pollinator not detected |
| Crib Point Cemetery | No plants  present | Damp woodland | -38.3548°, 145.1883° | 2018 | W1526, W1527, W1528 |
| Stoney Peak Rail Reserve | No plants present | Grassy  Woodland | 38.3731°, 145.2180° | 2018 | Present, not captured |
