# Supplementary material for: Sexual attraction with pollination during feeding behaviour: implications for transitions between specialized strategies
Source: Ann Bot. 2023 Nov 14;133(2):273–86. doi: 10.1093/aob/mcad178 (PMC11005785; doi:10.1093/aob/mcad178)
Supplement: mcad178_suppl_Supplementary_Data_S2 [file mcad178_suppl_supplementary_data_s2.docx]

**Supplement 2:** Location and voucher details for pollinator observations and floral dissections of species belonging to other species complexes of *Caladenia*. Plant vouchers representative of the source populations are lodged in the Western Australian Herbarium.

|  | Site name | Lat/long | Dates | Plant voucher |
| --- | --- | --- | --- | --- |
| *Caladenia attingens* | Holland Road; Fisher Road | 34°05'49.4"S 115°06'41.6"E;  34°15'35.0"S 115°11'29.4"E | 2,15,22/10/2014 | RDP 0281 PERTH 08646058 |
| *Caladenia crebra* | E Greenhead | 30°2′46.4″S; 115°03′20.2″E | 13-15/9/2014 | RDP 0426 PERTH 08978212 |
| *Caladenia infundibularis* | Fisher Road | 34°15'35.0"S 115°11'29.4"E | 11,17/10/2015; 27/10/2018 | RDP 0231 PERTH 08642974; RDP 0347 PERTH 08739625 |
| *Caladenia procera* | S Yallingup | 33°39ʹ32ʺS; 115°02ʹ09ʺE | 16-18/10, 25/10/2018 | RDP 0230 PERTH 08642966 |
