# Supplementary material for: Sexual attraction with pollination during feeding behaviour: implications for transitions between specialized strategies
Source: Ann Bot. 2023 Nov 14;133(2):273–86. doi: 10.1093/aob/mcad178 (PMC11005785; doi:10.1093/aob/mcad178)
Supplement: mcad178_suppl_Supplementary_Data_S4 [file mcad178_suppl_supplementary_data_s4.docx]

**Supplement 4**

*Methods*

We tested for statistical differences in the proportion of responding wasps that contacted the column, and the proportion of responding wasps that either fed or copulated with the flower using Generalized Linear Mixed Models (GLMM). These analyses were run in the R package lme4 using the function glmer with a binomial distribution. We treated experimental replicate (1–6) as a random factor in recognition that a different pair of flowers was used for each experimental replicate and the different environmental conditions that wasps were exposed to across experimental replicates. The statistical significance of the variable ‘site of odour release’ was tested by comparing the model with and without this variable using likelihood-ratio tests (using the function ‘anova’).

*Results*

**Figure 1**: Marginal means (expressed as predicted probability) at control and treatment flowers for: 1. Attempted copulation (*P* = <0.001), 2. Feeding (*P* = 0.137), and 3. Potential Pollination (*P* = 0.279).
